# Comparative Study of Machine Learning Approaches for Predicting Creep Behavior of Polyurethane Elastomer

**DOI:** 10.3390/polym13111768

**Published:** 2021-05-28

**Authors:** Chunhao Yang, Wuning Ma, Jianlin Zhong, Zhendong Zhang

**Affiliations:** School of Mechanical Engineering, Nanjing University of Science and Technology, Nanjing 210094, China; zhongjianlin@njust.edu.cn (J.Z.); zzd1157@163.com (Z.Z.)

**Keywords:** creep behavior, polyurethane elastomer, time–strain curve, machine learning, genetic algorithm

## Abstract

The long-term mechanical properties of viscoelastic polymers are among their most important aspects. In the present research, a machine learning approach was proposed for creep properties’ prediction of polyurethane elastomer considering the effect of creep time, creep temperature, creep stress and the hardness of the material. The approaches are based on multilayer perceptron network, random forest and support vector machine regression, respectively. While the genetic algorithm and *k*-fold cross-validation were used to tune the hyper-parameters. The results showed that the three models all proposed excellent fitting ability for the training set. Moreover, the three models had different prediction capabilities for the testing set by focusing on various changing factors. The correlation coefficient values between the predicted and experimental strains were larger than 0.913 (mostly larger than 0.998) on the testing set when choosing the reasonable model.

## 1. Introduction

Polymers are widely used in traditional industry, agriculture and high and new technology sectors due to their extensive sources, industrial maturity, and excellent properties (light weight, high strength, good toughness, etc.). Contemporary human life, from groceries to space shuttles and rockets, is closely related to polymers. In recent years, the service life of polymers has been required to be longer, even up to several decades of applications, which makes the long-term mechanical properties of polymers a hot research subject.

A polymer is a kind of substance with a polymer chain. The multiplicity, time dependence and temperature dependence of molecular chain movement makes polymer a typical viscoelastic material. Therefore, mechanical relaxation phenomena, such as the stress relaxation, creep and recovery of polymer is shown to be significant, and its mechanical behavior strongly depends on the time of exogenic force exertion [1]. The time dependence of viscoelastic material’s mechanical behavior indicates that there is characteristic time in the material [2]. The characteristic time is influenced by factors such as temperature [3], stress [4], strain [5] and physical aging [6]. The relative research shown that temperature could affect the characteristic time by changing the free volume of material [7]. For polymers, the effect of changing the temperature scale and time scale on their macro viscoelastic mechanical properties is equivalent [1]. Thus, the time–temperature superposition principle (TTSP) was presented. According to TTSP, the long-term mechanical properties of viscoelastic materials at lower temperatures can be obtained by shifting the short-term experimental curve at higher temperatures along the logarithmic time axis [1,7]. However, the time–stress superposition principle (TSSP) indicates that increasing the stress level has a similar effect [4]. These superposition principles make it possible to accelerate the characterization of the long-term mechanical properties of polymers [2].

Polyurethane elastomer (PUE) is an important type of polymer, which is made up of hard segment and soft segment, arranged alternately. Including isocyanate and chain extenders, the hard segment presents in glass state at room temperature, and has a lower glass transition temperature. However, the soft segment, polyol, presented in the hyper-elastic state at room temperature. The segments mentioned above provide the solid and elastic properties of the PUE [8]. Compared with the traditional rubber, PUE material has the advantages of high strength, a large adjustable range of performance, excellent wear resistance, oil resistance, ozone resistance, shock absorption, radiation resistance, air permeability and repeatable processing. Furthermore, PUE has been widely used in the automotive industry, bridge structures, marine structures as well as other important industries and consumer goods sectors [9].

Although the development of the long-term mechanical experiment of polymer is a direct method, it is also a simple and reliable way to verify the above superposition principles. However, the long-term mechanical experiment is much more time-consuming than the conventional mechanical experiment. The experiment is more difficult as the means to performing it are hard to obtain. Therefore, on the one hand, the superposition principles should be further expanded and improved continuously. On the other hand, accurate and efficient methods for the long-term mechanical properties of nonlinear viscoelastic material prediction should be sought in the field of machine learning, which has been developing rapidly in recent years. From this point of view, it has important theoretical significance and value to comprehensively understand and grasp the long-term mechanical properties of polymers, make full use of polymers and prevent accidents.

With the rapid development of machine learning technology, the artificial neural network (ANN) and support vector machine (SVM) are gradually being used in artificial intelligence and pattern recognition, such as system identification [10], predictive modeling [11], feedforward learning control [12] and fault diagnosis [13,14]. However, the application of the machine learning method to predict the nonlinear properties of materials is still in the initial stage. Based on the ANN, Rashidian and Hassanlourad [15] presented a model to predict the mechanical behavior of different carbonate soils. By inputting relative density, axial strain, maximum void ratio, calcium carbonate content and confining pressure, the deviatoric stress and volumetric strain at the end of each increment could be predicted. The accuracy and reliability of this method were verified by comparison with the experimental results. Based on the uniaxial compression experimental data, Shakiba et al. [16] developed the study of the relationship between the chemical composition, deformation variables and high-temperature flow behavior of Al–0.12Fe–0.1Si alloys using ANN model with 20 neurons in a hidden layer. The *k*-fold cross-validation method was used to evaluate the generalization capability of the model. Niu et al. [17] analyzed the prediction of a common rail direct injection system (CRDI)-assisted marine diesel engine using ANN and SVM methods. The taguchi orthogonal array was employed to obtain the test data, and high-precision prediction based on a small amount of training data was realized. The results show that the SVM model was well suited, while the ANN model fell into local minimum and over fitting. Qi et al. [18] employed genetic programming for the uniaxial compressive strength prediction of cemented paste backfill. The sampling method, training set size and maximum tree depth of the genetic programming performance was investigated. Using the relative variable frequency, partial dependence plots and relative importance scores, the relative variable importance was analyzed. The R${}^{2}$ of the testing set was larger than 0.8 after training. Rodriguez-Sanchez et al. [19] presented a feedforward ANN that was trained with stress/strain data of a thermoplastic elastomer. In contrast, five hyper-elastic models, the neural network model had the best accuracy with a coefficient of determination R${}^{2}$ = 0.996 and 1% difference from the experimental data. The ANN was combined with the nonlinear hyper-elastic finite element model to simulate the temperature-dependent stress response of elastomer solids in their following research [20]. Stoffel et al. [21] developed a series of ANN including a feedforward neural network, radial basis function neural network and a deep convolutional neural network to predict the structural deformations by comparison to experiments. Zhang et al. [22] proposed an intelligent agent model, based on the random forest (RF) and particle optimization swarm methods, to predict the long-term settlement creep index. The datasets of structural liquid limit, plasticity index, void ratio, clay content and creep index were collected through literature review. The RF model was established. Using the particle swarm optimization method and cross-validation method, the model was optimized. The results indicated that the prediction error of this method was significantly lower than that of existing empirical formulae. Then, the researchers developed a constitutive model for soils by using pure mathematical skills through learning from raw data using the machine learning method [23]. The research summarized the application of the machine learning algorithm in soil constitutive model development. The results showed that the long short-term memory neural network was most suitable for developing the constitutive model of the soil.

Similarly, several kinds of research have been carried out in the polymers field using machine learning techniques in recent years. According to the machine learning algorithm, Mannodi-Kanakkithodi et al. [24] developed an on-demand property prediction model to directly realize the design of polymers with given target properties using a genetic algorithm to evolutionarily optimize polymer constituent blocks. Then, by combining computations or experiments with machine learning techniques, they utilized a first principles-generated dataset of the electronic and dielectric properties of a chemical space of polymers to test different kinds of regression algorithms. Several possibilities for the hyper-parameters have been explored, and the optimal strategies and parameters for high-fidelity polymer dielectrics property prediction have been established [25]. Doan Tran et al. [26] provided an overview of some of the critical technical aspects based on the polymer genome machine learning method, including polymer data curation, representation, learning algorithms, and prediction model usage. Furthermore, the remaining challenges and possible future directions were discussed. Zhong et al. [27] built a long-term creep behavior prediction model of PMI materials using the ANN technique. The effects of different activation functions, hidden layer structures, and other super-parameters on the prediction performance were investigated. The results suggested that the statistical value of the correlation coefficient was greater than 0.995. Rahman et al. [28] developed a surrogate machine learning model trained with molecular dynamics models of functionalized CNT-epoxy and the corresponding interfacial shear strength. Yildirim et al. [29] predicted and compared perovskite solar cells performances, based on machine learning approaches, with those developed WO3 and its composites. The results showed that the decision tree model has a 0.9656 R${}^{2}$ score for the WO3-poly(3,4-ethylenedioxythiophene) and the random forest model has 0.9976, 0.9968, 0.9772 R${}^{2}$ scores for the WO3-poly(N-methylaniline), WO3-poly(2-fluoroaniline), and WO3-polyfuran, respectively. Yuan et al. [30] focused on the incomplete database of The Membrane Society of Australasia, by imputing missing values in the database using the machine learning method, which extended the potential use of the database.

In this paper, the feasibility of the machine learning method to predict the compression creep deformation of PUE with highly nonlinear properties was analyzed, when changing the conditions of creep time, temperature, stress and hardness of the material. Based on the experiment results, the multilayer perceptron (MLP) network, RF and SVM algorithms of machine learning were used, combining the genetic algorithm and cross-validation, the prediction model was established. The accuracy and stability of the model were studied by comparing the performance of training set fitting and new condition prediction of the three models. This paper opens up a new way to predict the long-term mechanical properties of polymers through the machine learning method, which could reduce the number of experiment working conditions as well as shorten the experiment period, and provide an idea for the accelerated characterization of long-term mechanical properties of materials, in addition to various superposition principles.

## 2. Materials and Methods

### 2.1. Creep Experiment

A commercial high-performance PUE produced by the casting machine of Nanjing Jinsanli Rubber & Plastic Co., Ltd. (Nanjing, China) was considered for a compression creep experiment in this research.

Traditional PUE material can be used for a long time under 80 °C, and its short-term service temperature is a maximum of 120 °C. It can be found that such low-temperature resistance limits its application. In this study, the polyurethane elastomer synthesized by 4,4′-methylenedianilin (MDI), polyether polyols, trihydroxymethylpropane crosslinking chain extender and auxiliaries were considered, which could improve the thermal stability and mechanical properties. The hardness of PUE was controlled by adjusting the component content.

**Figure 1:**
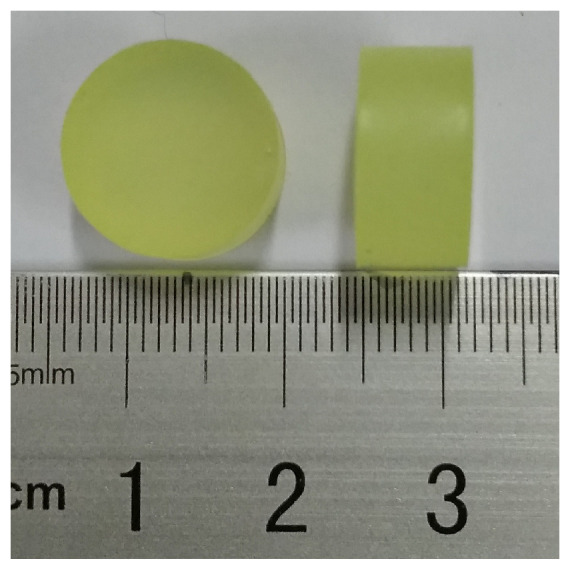
The material and specimens of PUE.

The cylindrical specimens with 12.5 mm diameter and 6.5 mm thickness were made by mold casting process, as shown in Figure 1. Each type of specimen was made of the same batch of raw materials and production, to ensure that the thermal and mechanical properties of specimens were consistent. The hyper-elastic properties of PUE were first proposed using a universal mechanical testing machine and other equipment. Three parallel experiments were conducted for each kind of hardness. The results are shown in Table 1.

**Figure 2:**
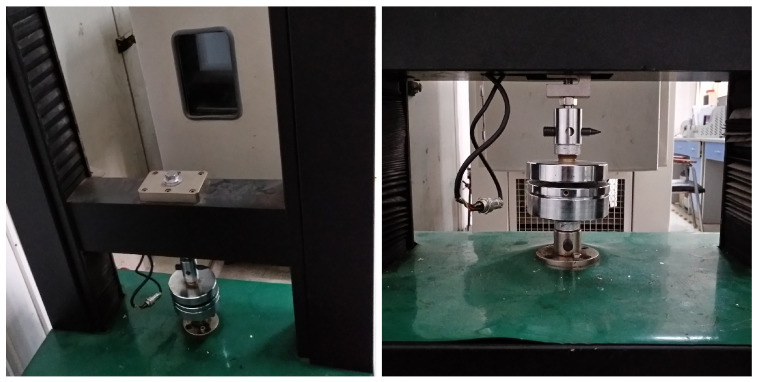
The universal mechanical testing machine with calorstat.

**Figure 3:**
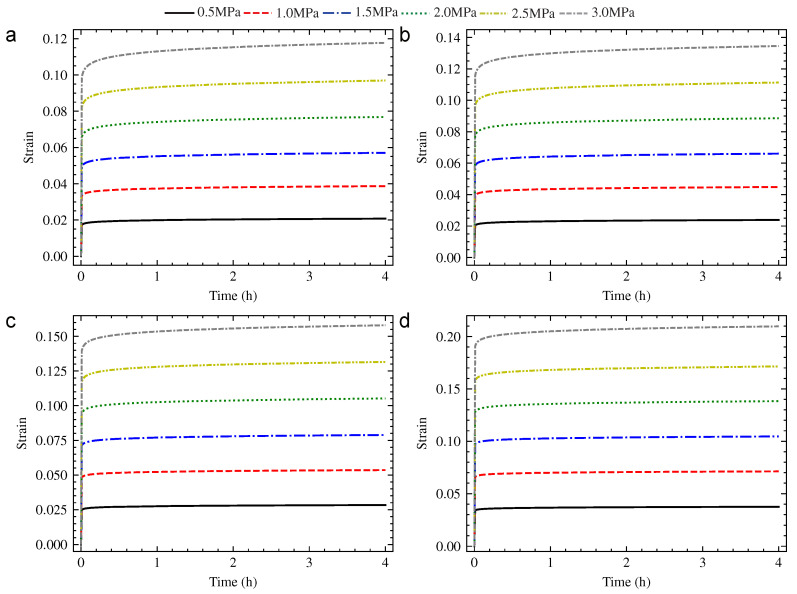
The strain–time creep curve of 70 HA PUE: (**a**) 20 °C; (**b**) 40 °C; (**c**) 65 °C; and (**d**) 90 °C.

**Figure 4:**
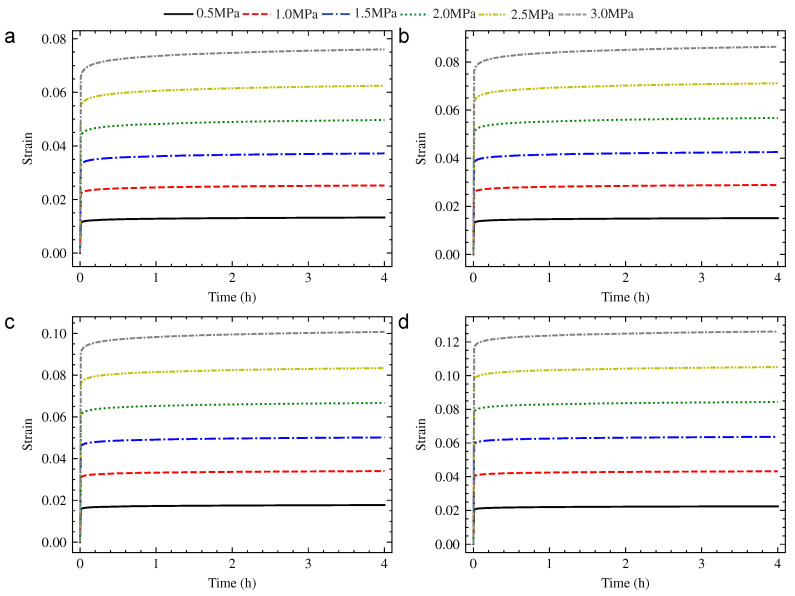
The strain–time creep curve of 80 HA PUE: (**a**) 20 °C; (**b**) 40 °C; (**c**) 65 °C; and (**d**) 90 °C.

**Figure 5:**
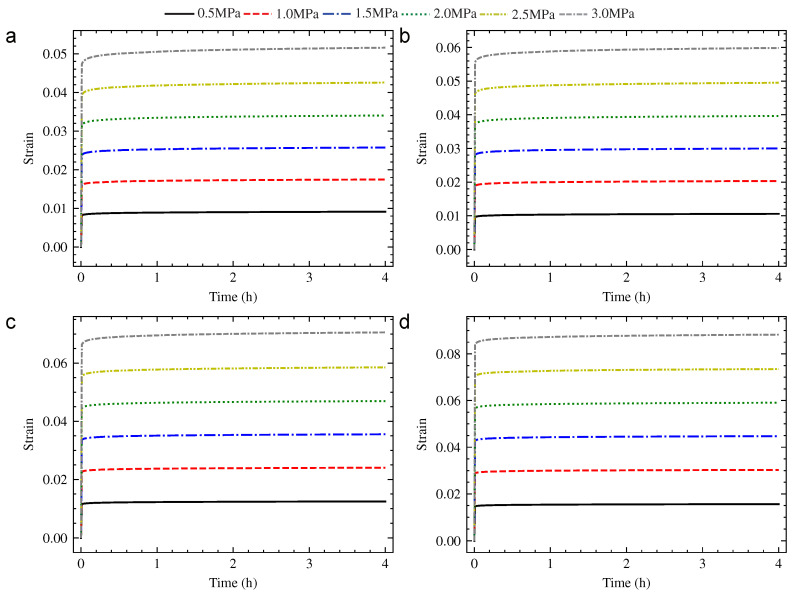
The strain–time creep curve of 90 HA PUE: (**a**) 20 °C; (**b**) 40 °C; (**c**) 65 °C; and (**d**) 90 °C.

The universal testing machine with calorstat was used to implement the compression creep experiment, as shown in Figure 2. The hardness of PUE, creep stress, creep temperature and creep time were considered as the research parameters. The hardness of PUE was 70 HA, 80 HA and 90 HA. The creep stress was 0.5 MPa, 1.0 MPa, 1.5 MPa, 2.0 MPa, 2.5 MPa and 3.0 MPa. The creep temperature was 20 °C, 40 °C, 65 °C and 90 °C. The standard creep time of the experiment was set to 4 h. The strain–time curve was obtained according to the compression creep experiment, as shown from Figure 3, Figure 4 and Figure 5.

The creep properties of the PUE material can be obtained by the equation:

(1) $$\varepsilon_{t}=\varepsilon_{e}+\varepsilon_{c}$$

where $\varepsilon_{t}$ refers to total strain, $\varepsilon_{e}$ refers to elastic strain and $\varepsilon_{c}$ refers to creep strain. The results were shown from Table 2, Table 3 and Table 4 by calculation. It could be seen that the creep strain $\varepsilon_{c}$ of PUE increased with the increase in creep stress and temperature at the same creep time. In the variable region of this research, the effect of creep stress was more significant.

### 2.2. Data Acquisition and Processing

#### 2.2.1. Input and Output Variables of the Model

The factors that affect the mechanical properties of polymer materials include time, stress, strain rate, temperature, humidity, aging, and crystallinity. From the results of the compression creep experiment mentioned in Section 2.1, creep stress and temperature had more influence on the creep properties of PUE. In addition, due to the different content of each component and their varying degree of crystallinity, when preparing the PUE, the hardness variance affects the macroscopic creep behavior of the material on the microscopic level. Therefore, the creep time $x_{ti}$, creep stress $x_{st}$, temperature $x_{te}$ and hardness of PUE $x_{h}$ were considered as the input variables $x={\left[x_{ti},x_{st},x_{te},x_{h}\right]}^{T}$. The axial strain of PUE was taken as the output variable. The range of input variables chosen from the experiment conditions is shown in Table 5.

#### 2.2.2. Data Analysis

**Figure 6:**
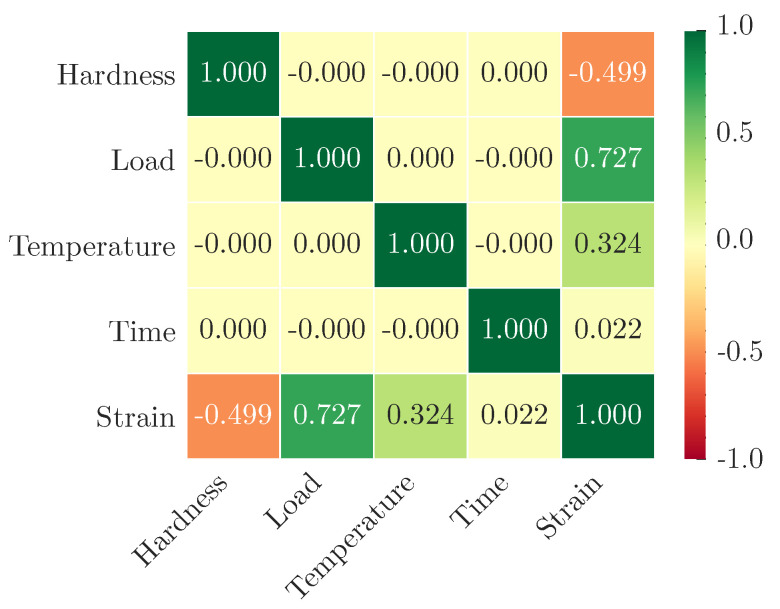
Correlation between the variables.

After determining the input and output variables, the correlation between the variables in the creep experiment results was analyzed, as shown in Figure 6.

It could be seen that the correlation coefficients between the input variables in this research were slim to zero, which means that the input variables were independent of each other. While there were varying degrees of correlation between the input and output variables. These results indicated that the input variables considered in this research are reasonable with no redundancy, and the research on the machine learning prediction model could be carried out on this basis.

#### 2.2.3. Data Normalization

The range of input variables and strain results of the creep experiment showed that the input and output variables were not in the same order of magnitude. However, multiple machine learning methods, such as ANN, require that the weights and other parameters in the model are parallel in order of magnitude. If the difference of input variables is large, the input variables with a smaller order of magnitude will be covered by those with a larger order of magnitude during the error propagation. Furthermore, the effect of each input variable on the output cannot be rendered properly. Consequently, normalizing the input and output variables is crucial before modeling.

In this research, the z-score method, which is commonly used alongside machine learning, was considered to normalize the input and output variables, so that the mean value of each variable equals 0 while the variance equals 1. The method can be expressed as

(2) $${\hat{x}}^{\left(n\right)}=\frac{x^{\left(n\right)}-\bar{x}}{S}$$

where $x^{\left(n\right)}$ are the original samples, n=1,2,⋯,N, and *N* is the number of samples, $\bar{x}$ is the mean value of samples, *S* is the variance of samples, ${\hat{x}}^{\left(n\right)}$ are the normalized samples. After normalization, the input variables can be expressed as $\hat{x}={\left[{\hat{x}}_{t},{\hat{x}}_{st},{\hat{x}}_{te},{\hat{x}}_{h}\right]}^{T}$, while the output variables change to $\hat{y}$.

### 2.3. Machine Learning Prediction Algorithms

#### 2.3.1. Multilayer Perceptron Network

**Figure 7:**
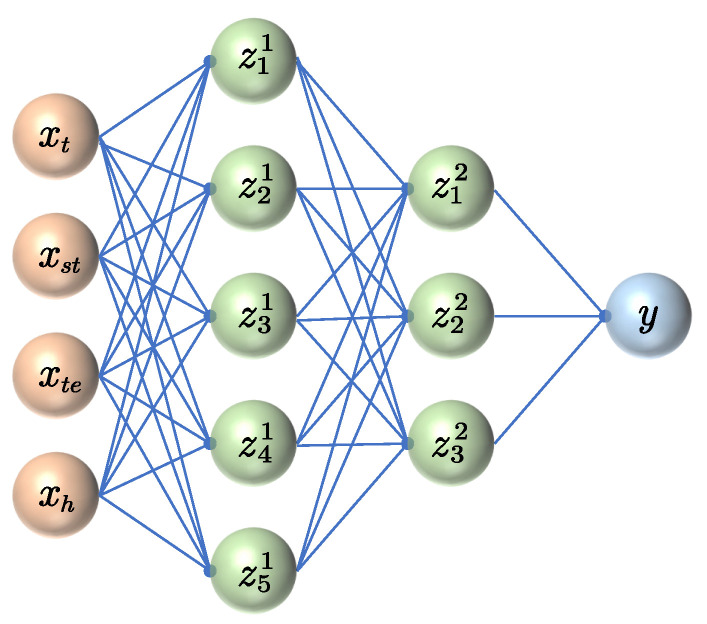
The structure of MLP network.

MLP network is a typical ANN, which is a nonlinear complex network system composed of a large number of “biological neurons”. As shown in Figure 7, a mathematical model is used to describe the biological neural network structure, so that the intelligent behavior to some extent can be simulated under the guidance of the algorithm. In this research, MLP based on the backpropagation algorithm is considered to train the prediction model. It is composed of an input layer, an output layer and at least one hidden layer. The training consists of two processes: signal forward propagation and error backpropagation. During the forward propagation, the input samples are transmitted from the input layer to each hidden layer and output layer. Then, the backpropagation stage begins if the output value is not equal to the real value. The error is allocated among all neural of the hidden layer by transmitting the output error back, and the error of neurons in each layer is obtained as the basis for optimizing the weights of neurons. The above processes are repeated until the output error is acceptable or reaches the training iterations’ limitation.

The nonlinear properties of the PUE creep strain–time curve were considered to have initially identified the main structural parameters range of the MLP network, as shown in Table 6. The activation function can be expressed as

(3) $$\mathrm{Logistic}\left(z\right)=\frac{1}{1+\exp(-z)}$$

(4) $$\mathrm{Tanh}\left(z\right)=\frac{\exp(z)-\exp(-z)}{\exp(z)+\exp(-z)}$$

(5) $$\mathrm{ReLU}\left(z\right)=\left\{\begin{array}{cc} z, & z\geq 0\\ 0, & z<0 \end{array}\right.$$

In order to avoid unnecessarily increasing the complexity of the model, the number of the hidden layer is set to 1 or 2. The number of neurons is setting from 1 to 100. The training method is selected from L-BFGS [31], SGD [32] and Adam [33]. Subsequently, the MLP network prediction model of PUE creep properties is constructed by optimizing the hyper-parameters of the model.

#### 2.3.2. Random Forest

**Figure 8:**
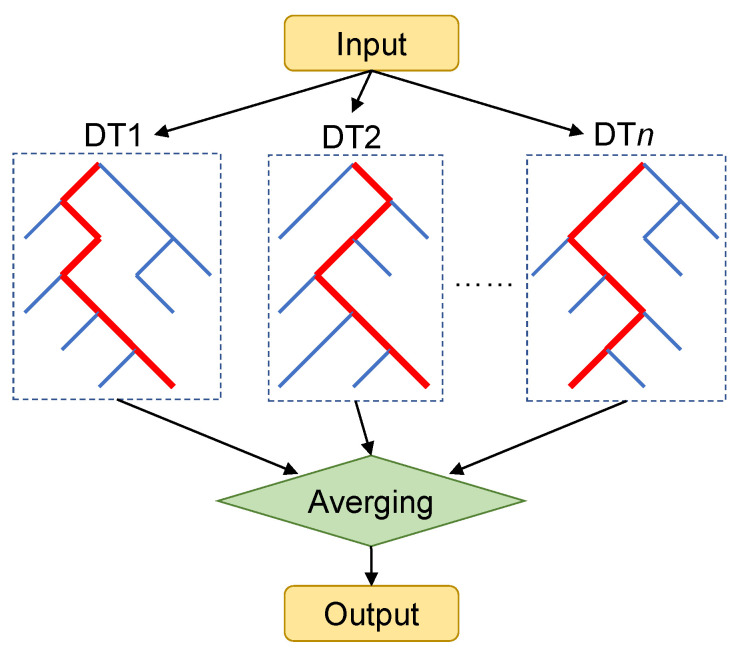
The structure of RF model.

RF is a machine learning algorithm that integrates multiple decision trees based on the idea of ensemble learning. Decision tree, the basic unit of RF, is a kind of tree-like structure with the function of data classification or regression. It is composed of an internal node, leaf node and directed edge, as shown in Figure 8. For the regression problem, the predictive value of each leaf node is the mean value of the training set elements’ output, which can be expressed as

(6) $$c_{m}=\mathrm{AVG}\left(y_{i}|x_{i}\in {\mathrm{leaf}}_{m}\right)$$

The leaf nodes represent the predicted value, and mean squared error (MSE) or mean absolute error (MAE) is generally used as the criteria to feature and split.

The main hyper-parameter range of RF was initially identified to include the maximum depth of RF (max depth), the maximum number of decision trees in the RF (max DT), the minimum number of samples at the leaf node (min samples leaf), and the minimum number of samples required to split an internal node (min samples split), as shown in Table 7. Subsequently, the RF prediction model of PUE creep properties is constructed by optimizing the hyper-parameters of the model.

#### 2.3.3. Support Vector Machine Regression

**Figure 9:**
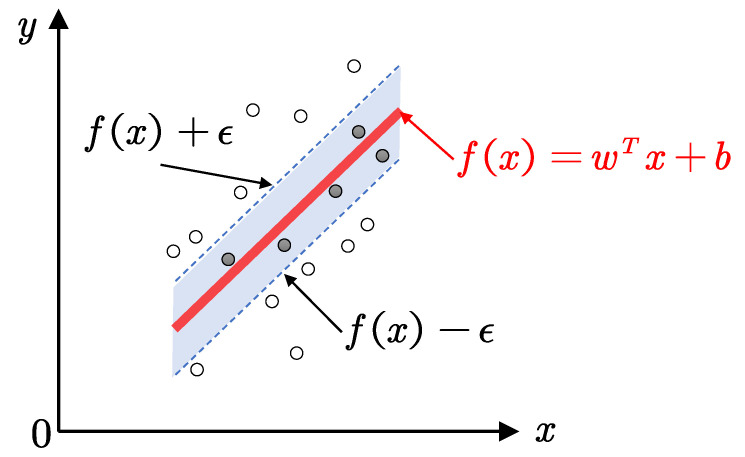
The method of SVR.

As one of the most common methods in the machine learning field, support vector machine regression (SVR) has shown its unique advantages in solving the problems of small sample, nonlinear and high-dimensional pattern recognition. SVR is developed from the optimal classification surface of a linearly separable problem, using nonlinear transformation defined by the inner product function to transform the sample input space into another higher dimensional space, and then solving the generalized optimal classification hyperplane. SVR mainly solves the finite sample problem and finds the best compromise between the complexity and the learning ability of the model in order to obtain the best generalization ability, as shown in Figure 9. The SVR method successfully avoids the traditional process from induction to deduction and efficiently realizes the “transductive inference” from training data to predicted data.

The basic idea of nonlinear SVR is to map the data x to the Hilbert feature space using a nonlinear mapping ϕ, then linear regression is carried out in this space. The kernel function $k\left(x_{i},x_{j}\right)=\phi \left(x_{i}\right)\cdot \phi \left(x_{j}\right)$ is used to realize the correspondence between the linear regression of high-dimensional space to the nonlinear regression of low-dimensional space. The SVR theory was widely introduced in formal research like Refs. [10,14], and a three-order RBF kernel was considered in this study. The main hyper-parameter range of SVR was initially identified to include C and gamma, as shown in Table 8.

#### 2.3.4. The Adjustment and Validation of Hyper-Parameters

**Figure 10:**
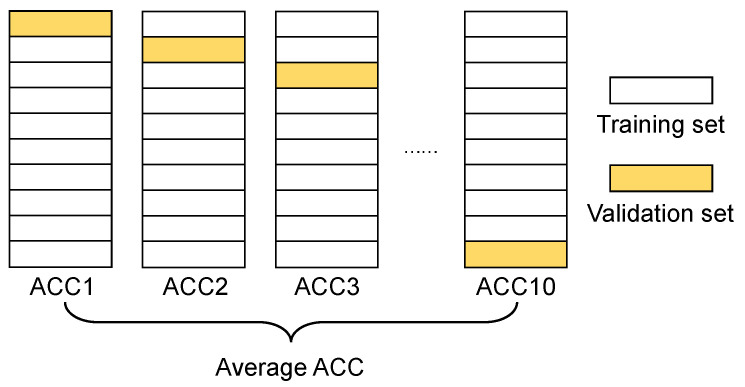
Te-fold cross-validation method.

In this research, the *k*-fold cross-validation method was used to avoid overfitting. The training set was randomly divided into *k* folds. The training set was composed of k−1 folds and the validation set was performed by the remaining fold. With various subsets being used as the validation set, the training process was repeated *k* times. The cross-validation error was obtained by averaging the MSE of *k* times. Then, the training accuracy of the model under the current hyper-parameters was represented. Ten-fold cross-validation was considered according to the number of experiment samples in this study, as shown in Figure 10.

The genetic algorithm was considered to optimize the hyper-parameters of the three machine learning models. The genetic algorithm mainly used the law of the “survival of the fittest” in the process of biological evolution, imitating the genetic reproduction mechanism. First, binary or other systems were used to code the individuals in the solution space of the optimization problem. Then, genetic operations such as selection, crossover and mutation are being carried out. By repeatedly and properly using the operators and selection principles of genetic algorithms, the population can continuously reproduce from parental generation to filial generation, which makes the adaptability of the population to the environment increase continuously. Through the iterative method mentioned above, the results with an optimal solution or better solution will be found from the new population. The parameters of the genetic algorithm in this research are shown in Table 9.

#### 2.3.5. Evaluation Index

**Figure 11:**
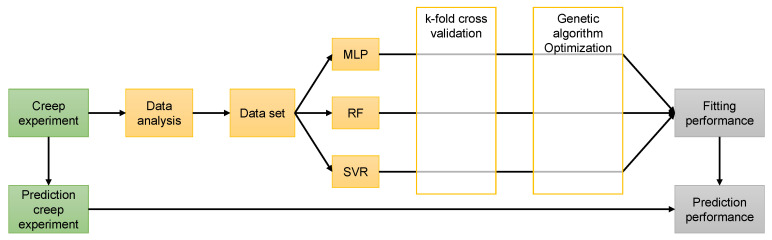
The scheme of building prediction model.

It is a typical regression problem to predict the creep properties of PUE. The evaluation index of the machine learning model of the regression problem includes the MAE, root mean square error (RMSE), correlation coefficient R and the coefficient of determination R${}^{2}$, which can be expressed as

(7) $$\mathrm{MAE}=\frac{1}{N}\sum_{i=1}^{N}\left|\left(y_{i}^{*}-{\hat{y}}_{i}\right)\right|$$

(8) $$\mathrm{RMSE}=\sqrt{\frac{1}{N}\sum_{i=1}^{N}{\left(y_{i}^{*}-{\hat{y}}_{i}\right)}^{2}}$$

(9) $$R=\frac{\mathrm{Cov}(x,y)}{\sqrt{\mathrm{Var}(x)\mathrm{Var}(y)}}$$

(10) $$R^{2}=1-\frac{\sum_{i}{\left(y_{i}^{*}-{\hat{y}}_{i}\right)}^{2}}{\sum_{i}{\left(y_{i}^{*}-\bar{y}\right)}^{2}}$$

where *N* is the number of samples, ${\hat{y}}_{i}$ is the normalization value of the *i*th sample, $y_{i}^{*}$ is the prediction value of the *i*th sample, and $\bar{y}$ is the mean value of samples, Cov(x,y) is the covariance, Var(x) is the variance. The four indicators mentioned above were considered to evaluate the ability of the three machine learning models. PYTHON 3.7 was used to build and train the machine learning models. The building method of prediction model in this research is shown in Figure 11.

## 3. Results and Discussion

### 3.1. Optimization Results of Hyper-Parameters

The cross-validation method and genetic algorithm mentioned in Section 2.3.4 were used to optimize the hyper-parameters of the MLP, RF and SVR machine learning prediction models. The results are shown as:

- MLP: activation function = logistic, number of hidden layers = 2, number of hidden layer neurons = 8, training method = Adam;
- RF: max depth = 8, max DT = 513; min samples leaf = 9; min samples split = 7;
- SVR: C = 4298, gamma = $7.2\times 10^{-4}$.

**Figure 12:**
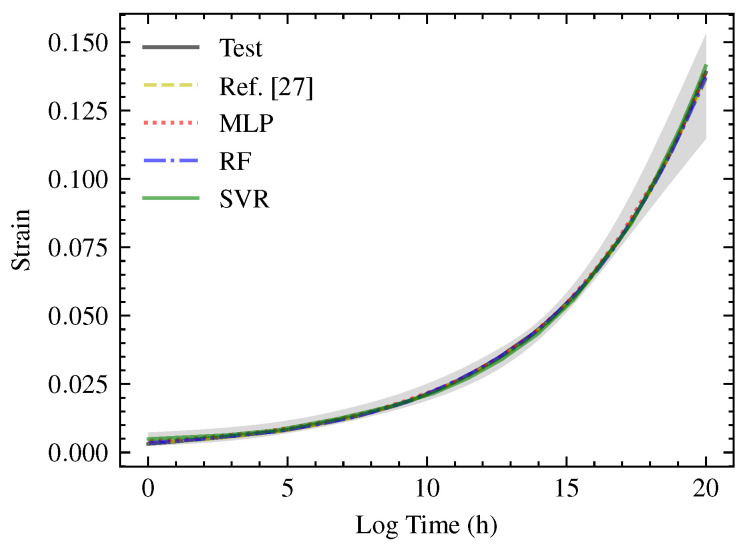
Comparison of the fitting and prediction performance with the results obtained from Ref. [27].

To verify the fitting and prediction performance of the model hyper-parameters above, the creep master curve data of PMI material in Ref. [27] were used for comparison. The creep curves at three temperatures were set to be the training set, and the creep curve at the other temperature was set to be the prediction set, respectively. Furthermore, 100 training groups were carried out. The results are shown in Figure 12.

The lines refer to the master curve processed by the test, Ref. [27], the model of present work, while the shaded area refers to the envelope range of the prediction after 100 training groups of Ref. [27]. It can be seen that the three machine learning models with optimized hyper-parameters have good fitting and prediction performance for the creep curve of materials and can be used for the creep performance prediction. Then, the strain–time curves of PUE creep predicted by the three machine learning methods mentioned above are shown in the next subsection. The overall trend, fitting performance of the training set and prediction performance of the prediction set were analyzed in detail.

### 3.2. Comparison of Fitting Performance of Training Set

**Figure 13:**
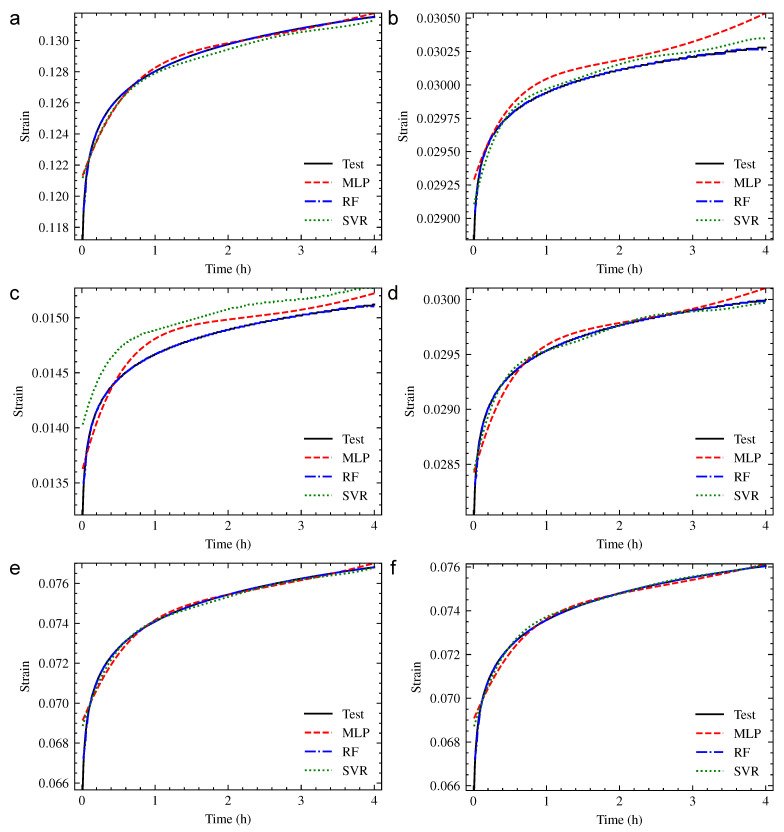
The fitting performance of each working condition: (**a**) Working Condition 1; (**b**) Working Condition 2; (**c**) Working Condition 3; (**d**) Working Condition 4; (**e**) Working Condition 5; (**f**) Working Condition 6.

Six compression creep working conditions of PUE were obtained by orthogonal experimental design method, as shown in Table 10. The fitting performance of the three machine learning methods was obtained through the contrast of model and the experimental strain–time creep curve, as shown in Figure 13. The index of fitting accuracy is shown in Table 11.

The fitting curve of the MLP model was basically consistent with the experiment curve under working conditions 1, 5 and 6. The fitting accuracy under working condition 4 was slightly decreased. The coefficient of determination R${}^{2}$ was 0.7443 and 0.9059 under conditions 2 and 3, respectively, which means that the fitting curve had a large deviation. The RF model had an excellent fitting performance under all six working conditions. The coefficient of determination R${}^{2}$ was larger than 0.997, which showed the best accuracy among the three methods. The fitting curve of the SVR model was basically consistent with the experiment curve under working conditions 1, 4, 5 and 6. The fitting accuracy under working condition 2 was slightly decreased. The coefficient of determination R${}^{2}$ was 0.5632, which is the lowest fitting accuracy of the three models.

By analyzing the fitting performance of the three models for the training set, it could be seen that the overall fitting performance of the RF model was the best. The accuracy of MLP and SVR models was sufficient in most working conditions, while numerical and trend distortion existed in some working conditions. In order to avoid the overfitting of current machine learning models, the prediction performance of PUE compression creeps under new working conditions was considered in Section 3.3.

### 3.3. Comparison of Prediction Performance

**Figure 14:**
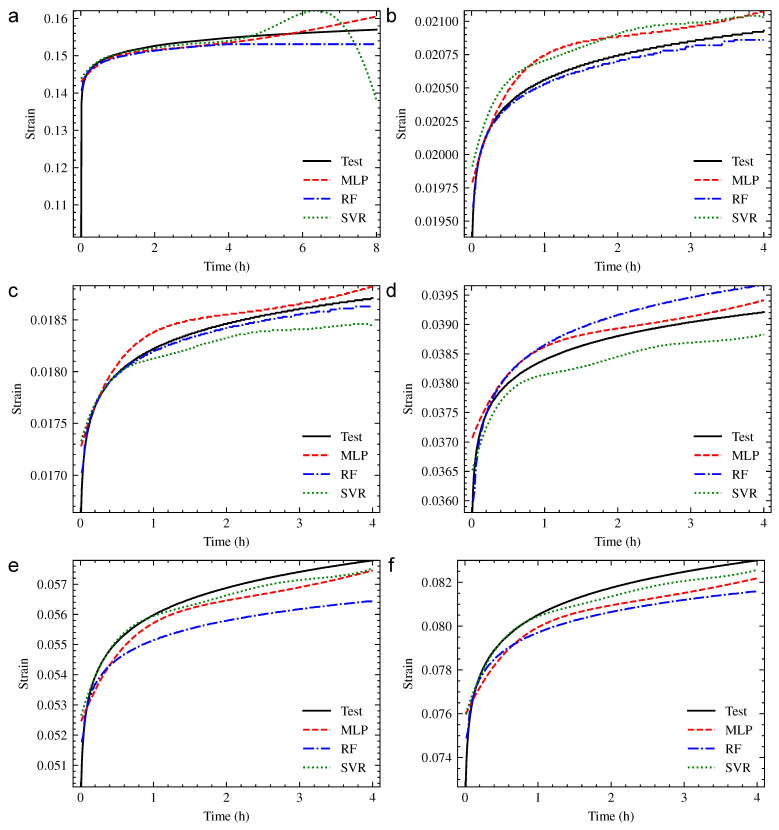
The prediction performance of each working condition: (**a**) Working Condition 1; (**b**) Working Condition 2; (**c**) Working Condition 3; (**d**) Working Condition 4; (**e**) Working Condition 5; (**f**) Working Condition 6.

Using the orthogonal experimental design method, the working conditions for machine learning prediction were designed by changing the experimental conditions in Table 10, as shown in Table 12. The changed conditions were written in bold. In this research, six prediction conditions were considered to carry out the compression creep experiment of PUE, and the prediction results of three machine learning models are shown in Figure 14. The prediction accuracy is shown in Table 13. The generalization ability of three prediction models was analyzed by changing the creep time, creep temperature, hardness of PUE and creep stress.

#### 3.3.1. Creep Time

According to the prediction results of working condition 1 shown in Figure 14a, when predicting 8 h creep properties of PUE, the prediction properties of 4 h prior were much better than 4 h later, due to the 4 h creep training set of models. In the interval of 4 h–8 h: there was a cross between the strain growth of the MLP model and the experiment curve, and the strain growth rate of prediction was higher than that of the experiment; the strain growth rate of RF model prediction was closest to that of the experiment, while the overall strain value was slightly smaller; the trend of SVR prediction was quite different from the experiment.

As mentioned in working condition 1 of Table 13, the evaluation index MAE, RMSE and R${}^{2}$ of the MLP model were better, while the evaluation index R of the RF model was better. The evaluation index of SVR was the worst among the three models. Therefore, the MLP and RF machine learning models have a better prediction ability than the SVR model in the working conditions of extending creep time.

#### 3.3.2. Creep Temperature

According to prediction results of working conditions 2 and 3 shown in Figure 14b,c, when predicting the creep properties of PUE in the range of 20 °C–90 °C, using the three machine learning models: the prediction creep curves of the RF model were the closest to the experiment curves; the prediction curves of the MLP model under two temperatures were both higher than the experiment curves, and the overall trend was nearly the same, while the prediction accuracy was worse than that of the RF model; the prediction ability of the SVR model represented instability, and the bug contrast of experiment curves, the prediction curve was higher under 30 °C and lower under 50 °C, which had the worst overall trend among the three models.

As mentioned in working conditions 2 and 3 of Table 13, the four evaluation indexes of the RF model were the best, which means the prediction accuracy was the best. The MAE, RMSE and R${}^{2}$ of the MLP model were better than that of the SVR model, while the evaluation index R of the SVR model was better. Therefore, the main priority of machine learning methods under the working conditions of the changing creep temperature of PUE was RF.

#### 3.3.3. Hardness of PUE

According to prediction results of working condition 4 shown in Figure 14d, when predicting creep properties of PUE by changing the hardness: the prediction creep curves of the MLP model were the closest to the experiment curves, and the trend was nearly the same, which indicates the highest prediction accuracy; the prediction curves of the RF model had the nearest overall trend with the experiment curve, while the curve value was higher than that of the experiment, and the accuracy was worse than the MLP model. The overall trend of the SVR model was the worst among the three models, and the curve value was lower than that of the experiment.

As mentioned in working condition 4 of Table 13, the evaluation index MAE, RMSE and R${}^{2}$ of the MLP model were the best, while the evaluation index R of the RF model was the best. Therefore, the main priority of the machine learning methods in the working conditions of changing the hardness of PUE was MLP.

#### 3.3.4. Creep Stress

According to the prediction results of working conditions 5 and 6 shown in Figure 14e,f, when changing the creep stress of PUE: the prediction creep curves of the SVR model were the closest to the experiment curves, and MLP model came second. The overall trend of creep curves predicted by the three models was all close to the experiment curve.

As mentioned in working conditions 5 and 6 of Table 13, the evaluation index MAE, RMSE and R${}^{2}$ of the SVR model were the best, while the evaluation index R of the RF model was the best. Therefore, the main priority of the machine learning methods in the working conditions of changing the creep stress was SVR.

### 3.4. Limitations

Although a new idea was put forward for the prediction of compression creep properties of polymer materials, the author thinks that the current research still has the following limitations:

- The experimental working conditions considered were few and the total dataset was relatively small. The accuracy and reliability of the machine learning models such as MPL, RF and SVR would be enhanced under a larger dataset;
- Only four creep relative variables including creep time, temperature, hardness of the material and creep stress were considered. However, the creep properties of polymer materials are more complicated. Therefore, more variables should be involved in the subsequent research to further optimize the prediction model;
- Due to the inconsistency of the specimen’s size, thermal properties of material and experiment error, the inherent laws of the samples under different working conditions were weakened, while the nonlinear properties were further intensified. The training difficulty of the prediction model was increased and the prediction accuracy was reduced.

## 4. Conclusions

In this research, a series of machine learning methods (based on MLP, RF and SVR) was used to predict the compressive creep deformation of PUE materials. Considering the variables of creep time, creep temperature, hardness of the material and creep stress, the compression creeps experiment of the PUE specimen was carried out. The genetic algorithm and *k*-fold cross-validation method were used to optimize the hyper-parameters of the model, and the fitting accuracy of the model in the training set and the prediction ability under new working conditions were verified. According to the results, the following conclusions could be obtained:

- The creep properties of PUE was closely related to creep time, creep temperature, creep stress and hardness, which showed strong nonlinear characteristics;
- The optimization method by combining genetic algorithm and *k*-fold cross-validation to the machine learning model’s hyper-parameters could effectively improve the fitting accuracy in the training set;
- The generalization ability of the MLP model was better when changing the creep time and hardness of the material while changing the creep temperature and creep stress was relatively poor;
- The generalization ability of the RF model was better when changing the creep time, creep temperature and hardness of material, while changing the creep stress was relatively poor;
- The generalization ability of the SVR model was better when changing the creep stress while changing the creep time, creep temperature and hardness of material were relatively poor;
- The method described in this research was the application case of machine learning technology in the field of mechanical response analysis, which could provide a new research idea for the accelerated representation of long-term mechanical properties of polymers.

## Figures and Tables

**Table 1:** The hyper-elastic properties of PUE.

Hardness (HA)	70	80	90
Linear elastic modulus (MPa)	15.87	36.26	55.14
Hardening stress (MPa)	9.15	21.05	30.81
Hardening strain	0.39	0.41	0.42

**Table 2:** Compression creep properties of 70 HA PUE.

Creep Stress (MPa)		0.5	1.0	1.5	2.0	2.5	3.0
	$\varepsilon_{e}$ (%)	1.513	3.003	4.471	5.917	7.342	8.746
20 °C	$\varepsilon_{t}$ (%)	2.074	3.868	5.705	7.681	9.692	11.771
	$\varepsilon_{c}$ (%)	0.561	0.865	1.234	1.764	2.350	3.025
	$\varepsilon_{e}$ (%)	1.818	3.613	5.376	7.107	8.808	10.390
40 °C	$\varepsilon_{t}$ (%)	2.387	4.482	6.608	8.859	11.131	13.457
	$\varepsilon_{c}$ (%)	0.569	0.869	1.232	1.752	2.323	3.067
	$\varepsilon_{e}$ (%)	2.258	4.484	6.652	8.761	10.843	12.714
65 °C	$\varepsilon_{t}$ (%)	2.834	5.356	7.887	10.521	13.153	15.801
	$\varepsilon_{c}$ (%)	0.576	0.872	1.235	1.760	2.310	3.087
	$\varepsilon_{e}$ (%)	3.165	6.249	9.218	12.051	14.818	17.856
90 °C	$\varepsilon_{t}$ (%)	3.746	7.125	10.46	13.828	17.144	20.968
	$\varepsilon_{c}$ (%)	0.581	0.876	1.242	1.777	2.326	3.112

**Table 3:** Compression creep properties of 80 HA PUE.

Creep Stress (MPa)		0.5	1.0	1.5	2.0	2.5	3.0
	$\varepsilon_{e}$ (%)	1.019	2.028	3.027	4.016	4.995	5.964
20 °C	$\varepsilon_{t}$ (%)	1.328	2.525	3.721	4.968	6.247	7.605
	$\varepsilon_{c}$ (%)	0.309	0.497	0.694	0.952	1.252	1.641
	$\varepsilon_{e}$ (%)	1.193	2.382	3.557	4.718	5.855	6.979
40 °C	$\varepsilon_{t}$ (%)	1.512	2.887	4.257	5.673	7.116	8.632
	$\varepsilon_{c}$ (%)	0.319	0.505	0.700	0.955	1.261	1.653
	$\varepsilon_{e}$ (%)	1.444	2.887	4.309	5.701	7.062	8.405
65 °C	$\varepsilon_{t}$ (%)	1.772	3.399	5.013	6.665	8.337	10.072
	$\varepsilon_{c}$ (%)	0.328	0.512	0.704	0.964	1.275	1.667
	$\varepsilon_{e}$ (%)	1.904	3.801	5.653	7.459	9.221	10.940
90 °C	$\varepsilon_{t}$ (%)	2.240	4.319	6.367	8.436	10.507	12.622
	$\varepsilon_{c}$ (%)	0.336	0.518	0.714	0.977	1.286	1.682

**Table 4:** Compression creep properties of 90 HA PUE.

Creep Stress (MPa)		0.5	1.0	1.5	2.0	2.5	3.0
	$\varepsilon_{e}$ (%)	0.757	1.508	2.254	2.994	3.729	4.458
20 °C	$\varepsilon_{t}$ (%)	0.913	1.746	2.574	3.402	4.255	5.157
	$\varepsilon_{c}$ (%)	0.156	0.238	0.320	0.408	0.526	0.699
	$\varepsilon_{e}$ (%)	0.892	1.785	2.671	3.549	4.419	5.281
40 °C	$\varepsilon_{t}$ (%)	1.058	2.033	3.000	3.964	4.951	5.984
	$\varepsilon_{c}$ (%)	0.166	0.248	3.216	4.272	5.316	6.349
	$\varepsilon_{e}$ (%)	1.071	2.149	3.216	4.272	5.316	6.349
65 °C	$\varepsilon_{t}$ (%)	1.246	2.406	3.553	4.694	5.853	7.055
	$\varepsilon_{c}$ (%)	0.175	0.257	0.337	0.422	0.537	0.706
	$\varepsilon_{e}$ (%)	1.376	2.763	4.131	5.474	6.807	8.112
90 °C	$\varepsilon_{t}$ (%)	1.562	3.028	4.475	5.907	7.349	8.824
	$\varepsilon_{c}$ (%)	0.186	0.265	0.344	0.433	0.542	0.712

**Table 5:** Range of input variables.

Time (h)	Stress (MPa)	Temperature (°C)	Hardness (HA)
[0,4]	0.5–3.0	20–90	70–90

**Table 6:** The range of MLP network structure hyper-parameters.

Hyper-Parameters	Range
Activation function	Logistic, Tanh, Relu
Number of hidden layers	1–2
Number of hidden layer neurons	1–10
Training method	L-BFGS, SGD, Adam

**Table 7:** The range of RF hyper-parameters.

Hyper-Parameters	Range
max depth	1–20
max DT	1–1000
min samples leaf	1–10
min samples split	2–10

**Table 8:** The range of SVR hyper-parameters.

Hyper-Parameters	Range
C	1–5000
gamma	[$1\times 10^{-5}$, 0.1]

**Table 9:** The parameters of genetic algorithm.

Parameters	Value
Number of chromosomes	1000
Number of generations	200
Genetic possibility	Crossover (90%), mutation (1%)
Fitness function	Correlation coefficient
Selection method	Tournament (size = 3)

**Table 10:** The creep working conditions for fitting.

Working Conditions	Hardness (HA)	Creep Stress (MPa)	Temperature (°C)	Creep Time (h)
1	70	2.5	65	4
2	90	1.0	90	4
3	80	0.5	40	4
4	90	1.5	40	4
5	70	2.0	20	4
6	80	3.0	20	4

**Table 11:** The training performance of three machine learning models.

Working Conditions	Model	MAE	RMSE	R	R${}^{2}$
	MLP	$1.860\times 10^{-4}$	$3.518\times 10^{-4}$	0.9892	0.9783
1	RF	$1.492\times 10^{-5}$	$1.086\times 10^{-4}$	0.9990	0.9979
	SVR	$3.000\times 10^{-4}$	$3.923\times 10^{-4}$	0.9925	0.9730
	MLP	$1.056\times 10^{-4}$	$1.192\times 10^{-4}$	0.9810	0.7443
2	RF	$2.275\times 10^{-6}$	$1.207\times 10^{-5}$	0.9987	0.9974
	SVR	$4.178\times 10^{-5}$	$4.692\times 10^{-5}$	0.9945	0.9604
	MLP	$8.710\times 10^{-5}$	$9.563\times 10^{-5}$	0.9848	0.9059
3	RF	$2.075\times 10^{-6}$	$1.585\times 10^{-5}$	0.9987	0.9974
	SVR	$1.966\times 10^{-4}$	$2.060\times 10^{-4}$	0.9946	0.5632
	MLP	$4.720\times 10^{-5}$	$6.440\times 10^{-5}$	0.9890	0.9595
4	RF	$2.225\times 10^{-6}$	$1.585\times 10^{-5}$	0.9988	0.9975
	SVR	$2.405\times 10^{-5}$	$3.606\times 10^{-5}$	0.9939	0.9873
	MLP	$1.372\times 10^{-4}$	$2.884\times 10^{-4}$	0.9879	0.9758
5	RF	$1.075\times 10^{-5}$	$8.598\times 10^{-5}$	0.9989	0.9978
	SVR	$1.080\times 10^{-4}$	$2.419\times 10^{-4}$	0.9927	0.9830
	MLP	$1.273\times 10^{-4}$	$2.724\times 10^{-4}$	0.9874	0.9743
6	RF	$1.065\times 10^{-5}$	$7.987\times 10^{-5}$	0.9989	0.9978
	SVR	$7.692\times 10^{-5}$	$2.156\times 10^{-4}$	0.9928	0.9839

**Table 12:** The creep working conditions for prediction.

Working Conditions	Hardness (HA)	Creep Stress (MPa)	Temperature (°C)	Creep Time (h)
1	70	2.5	65	**8**
2	90	1.0	**30**	4
3	80	0.5	**50**	4
4	**85**	1.5	40	4
5	70	**1.25**	20	4
6	80	**2.75**	20	4

**Table 13:** The generalization ability of three machine learning models.

Working Conditions	Model	MAE	RMSE	R	R${}^{2}$
	MLP	$1.233\times 10^{-3}$	$2.033\times 10^{-3}$	0.8424	0.6663
1	RF	$2.110\times 10^{-3}$	$2.692\times 10^{-3}$	0.9132	0.4151
	SVR	$2.339\times 10^{-3}$	$4.20\times 10^{-3}$	0.5260	−0.4251
	MLP	$1.294\times 10^{-4}$	$1.374\times 10^{-4}$	0.9851	0.7058
2	RF	$3.807\times 10^{-5}$	$4.139\times 10^{-54}$	0.9987	0.9733
	SVR	$1.486\times 10^{-4}$	$1.516\times 10^{-4}$	0.9939	0.6416
	MLP	$9.292\times 10^{-5}$	$1.073\times 10^{-4}$	0.9877	0.8994
3	RF	$4.115\times 10^{-5}$	$4.874\times 10^{-5}$	0.9986	0.9792
	SVR	$1.417\times 10^{-4}$	$1.612\times 10^{-4}$	0.9941	0.7730
	MLP	$1.588\times 10^{-4}$	$1.839\times 10^{-4}$	0.9892	0.8921
4	RF	$3.263\times 10^{-4}$	$3.468\times 10^{-4}$	0.9988	0.6163
	SVR	$3.123\times 10^{-4}$	$3.223\times 10^{-4}$	0.9938	0.6688
	MLP	$4.144\times 10^{-4}$	$4.378\times 10^{-4}$	0.9885	0.8759
5	RF	$9.956\times 10^{-4}$	$1.044\times 10^{-3}$	0.9989	0.2946
	SVR	$2.189\times 10^{-4}$	$2.857\times 10^{-4}$	0.9936	0.9471
	MLP	$7.816\times 10^{-4}$	$8.142\times 10^{-4}$	0.9876	0.7726
6	RF	$1.005\times 10^{-3}$	$1.066\times 10^{-3}$	0.9988	0.6103
	SVR	$3.272\times 10^{-4}$	$4.215\times 10^{-4}$	0.9931	0.9391

## Data Availability

Not applicable.

## References

[1] Shaw M.T., MacKnight W.J. (2005). Introduction to Polymer Viscoelasticity.

[2] Luo W., Wang C., Hu X., Yang T. (2012). Long-term creep assessment of viscoelastic polymer by time-temperature-stress superposition. Acta Mech. Solida Sin..

[3] Denardin E., Janissek P., Samios D. (2002). Time–temperature dependence of the thermo-oxidative aging of polychloroprene rubber. Thermochim. Acta.

[4] Brostow W. (2000). Time-stress correspondence in viscoelastic materials: An equation for the stress and temperature shift factor. Mater. Res. Innov..

[5] Wortmann F., Schulz K. (1995). Stress relaxation and time/temperature superposition of polypropylene fibres. Polymer.

[6] Struik L.C.E. (1976). Physical aging in amorphous glassy polymers. Ann. N. Y. Acad. Sci..

[7] He M., Qi X., Liu X., Su C., Lv N. (2015). Estimating the viscosity of pure refrigerants and their mixtures by free-volume theory. Int. J. Refrig..

[8] Bajsić E.G., Filipan V., Bulatović V.O., Mandić V. (2017). The influence of filler treatment on the mechanical properties and phase behavior of thermoplastic polyurethane/polypropylene blends. Polym. Bull..

[9] Engels H.W., Pirkl H.G., Albers R., Albach R.W., Krause J., Hoffmann A., Casselmann H., Dormish J. (2013). Polyurethanes: Versatile Materials and Sustainable Problem Solvers for Today’s Challenges. Angew. Chem. Int. Ed..

[10] Song J., Takakura H., Okabe Y., Kwon Y. (2009). Unsupervised Anomaly Detection Based on Clustering and Multiple One-Class SVM. IEICE Trans. Commun..

[11] Qi J., Hu J., Peng Y., Ren Q., Wang W., Zhan Z. (2011). Integration of similarity measurement and dynamic SVM for electrically evoked potentials prediction in visual prostheses research. Expert Syst. Appl..

[12] Sun Z., Yang X., Sun Y. Feedforward control based on the particle filter realization of SVM. Proceedings of the 2004 IEEE International Conference on Systems, Man and Cybernetics (IEEE Cat. No.04CH37583).

[13] He Z., Fu L., Lin S., Bo Z. (2010). Fault Detection and Classification in EHV Transmission Line Based on Wavelet Singular Entropy. IEEE Trans. Power Deliv..

[14] Liu J., Li Y.F., Zio E. (2017). A SVM framework for fault detection of the braking system in a high speed train. Mech. Syst. Signal Process..

[15] Rashidian V., Hassanlourad M. (2014). Application of an Artificial Neural Network for Modeling the Mechanical Behavior of Carbonate Soils. Int. J. Geomech..

[16] Shakiba M., Parson N., Chen X.G. (2016). Modeling the effects of Cu content and deformation variables on the high-temperature flow behavior of dilute Al-Fe-Si alloys using an artificial neural network. Materials.

[17] Niu X., Yang C., Wang H., Wang Y. (2017). Investigation of ANN and SVM based on limited samples for performance and emissions prediction of a CRDI-assisted marine diesel engine. Appl. Therm. Eng..

[18] Qi C., Fourie A., Ma G., Tang X., Du X. (2018). Comparative Study of Hybrid Artificial Intelligence Approaches for Predicting Hangingwall Stability. J. Comput. Civ. Eng..

[19] Rodriguez-Sanchez A.E., Ledesma-Orozco E., Ledesma S., Vidal-Lesso A. (2019). Application of artificial neural networks to map the mechanical response of a thermoplastic elastomer. Mater. Res. Express.

[20] Rodríguez-Sánchez A.E., Ledesma S., Vidal-Lesso A., Ledesma-Orozco E. (2020). The use of neural networks and nonlinear finite element models to simulate the temperature-dependent stress response of thermoplastic elastomers. Proc. Inst. Mech. Eng. Part L J. Mater. Des. Appl..

[21] Stoffel M., Gulakala R., Bamer F., Markert B. (2020). Artificial neural networks in structural dynamics: A new modular radial basis function approach vs. convolutional and feedforward topologies. Comput. Methods Appl. Mech. Eng..

[22] Zhang P., Yin Z.Y., Jin Y.F., Chan T.H. (2020). A novel hybrid surrogate intelligent model for creep index prediction based on particle swarm optimization and random forest. Eng. Geol..

[23] Zhang P., Yin Z.Y., Jin Y.F. (2021). State-of-the-Art Review of Machine Learning Applications in Constitutive Modeling of Soils. Arch. Comput. Methods Eng..

[24] Mannodi-Kanakkithodi A., Pilania G., Huan T.D., Lookman T., Ramprasad R. (2016). Machine Learning Strategy for Accelerated Design of Polymer Dielectrics. Sci. Rep..

[25] Mannodi-Kanakkithodi A., Pilania G., Ramprasad R. (2016). Critical assessment of regression-based machine learning methods for polymer dielectrics. Comput. Mater. Sci..

[26] Doan Tran H., Kim C., Chen L., Chandrasekaran A., Batra R., Venkatram S., Kamal D., Lightstone J.P., Gurnani R., Shetty P. (2020). Machine-learning predictions of polymer properties with Polymer Genome. J. Appl. Phys..

[27] Zhong J., Yang C., Ma W., Zhang Z. (2021). Long-term creep behavior prediction of polymethacrylimide foams using artificial neural networks. Polym. Test..

[28] Rahman A., Deshpande P., Radue M.S., Odegard G.M., Gowtham S., Ghosh S., Spear A.D. (2021). A machine learning framework for predicting the shear strength of carbon nanotube-polymer interfaces based on molecular dynamics simulation data. Compos. Sci. Technol..

[29] Yildirim M.O., Gok E.C., Hemasiri N.H., Eren E., Kazim S., Oksuz A.U., Ahmad S. (2021). A Machine Learning Approach for Metal Oxide Based Polymer Composites as Charge Selective Layers in Perovskite Solar Cells. ChemPlusChem.

[30] Yuan Q., Longo M., Thornton A.W., McKeown N.B., Comesaña-Gándara B., Jansen J.C., Jelfs K.E. (2021). Imputation of missing gas permeability data for polymer membranes using machine learning. J. Membr. Sci..

[31] Morales J.L., Nocedal J. (2011). Remark on algorithm 778: L-BFGS-B: Fortran subroutines for large-scale bound constrained optimization. ACM Trans. Math. Softw..

[32] Xu W. (2011). Towards Optimal One Pass Large Scale Learning with Averaged Stochastic Gradient Descent. arXiv.

[33] Kingma D.P., Ba J.L. (2014). Adam: A method for stochastic optimization. arXiv.

